# Supplementary material for: Environmental RNA interference in two-spotted spider mite, Tetranychus urticae, reveals dsRNA processing requirements for efficient RNAi response
Source: Sci Rep. 2020 Nov 5;10:19126. doi: 10.1038/s41598-020-75682-6 (PMC7644771; doi:10.1038/s41598-020-75682-6)
Supplement: Supplementary file 1 — Supplementary Information. [file 41598_2020_75682_MOESM1_ESM.pdf]

Supplementary Information

Environmental RNA interference in two-spotted spider mite, *Tetranychus urticae,* reveals dsRNA processing requirements for efficient RNAi response

Nicolas Bensoussan[1], Sameer Dixit[1], Midori Tabara[2,4], David Letwin[1], Maja Milojevic[1], Michele Antonacci[1], Pengyu Jin[1], Yuka Arai[3], Kristie Bruinsma[1], Takeshi Suzuki[2,3,4], Toshiyuki Fukuhara[2,4], Vladimir Zhurov[1], Sven Geibel[5], Ralf Nauen[5], Miodrag Grbic[1,6,7,*], Vojislava Grbic[1,6,*]

[1]Department of Biology, The University of Western Ontario, London, Ontario, N6A 5B8, Canada. [2]Department of Applied Biological Science, Tokyo University of Agriculture and Technology, Fuchu, Tokyo, 183-8509, Japan. [3]Graduate School of Bio-Applications and Systems Engineering, Tokyo University of Agriculture and Technology, Koganei, Tokyo, 184-8588 Japan. [4]Institute of Global Innovation Research, Tokyo University of Agriculture and Technology, Fuchu, Tokyo, 183-8509, Japan. [5]Division Crop Science, Research and Development, Bayer AG, Monheim, Germany. [6]Instituto de Ciencias de la Vid y el Vino, Logrono, 26006, Spain. [7]Department of Biology, University of Belgrade, Belgrade, 11000, Serbia.
*corresponding authors; e-mails: vgrbic@uwo.ca; mgrbic@uwo.ca

Supplemental Figure 1: Survival curves of adult female mites after treatment with a second non-overlapping dsRNAs targeting *T. urticae* orthologs of *Tribolium* RNAi-sensitive genes. Survival curves were plotted using the Kaplan-Meier method and compared using the log-rank test with Bonferroni correction (not significant, $P > 0.05$; **, $P < 0.01$; ***, $P < 0.001$).

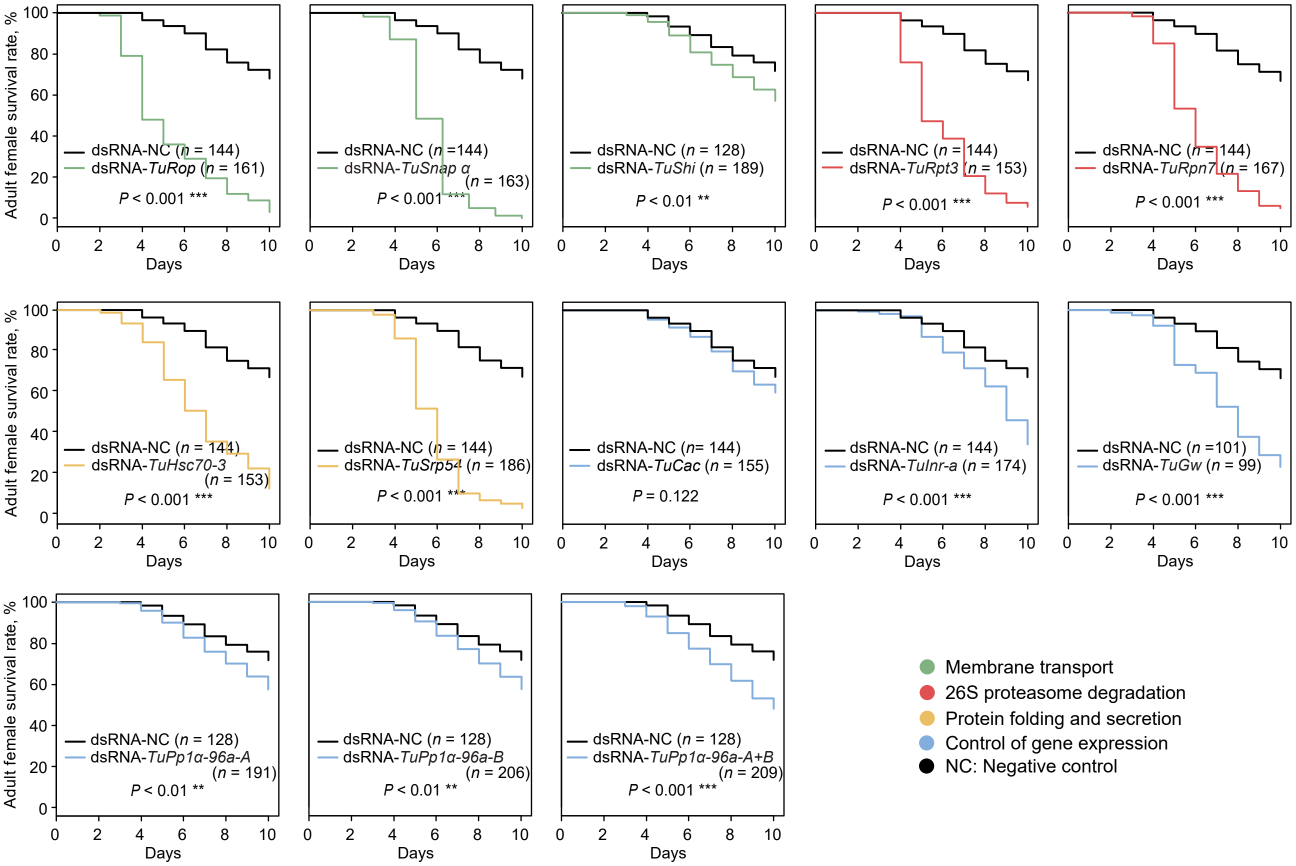

Supplemental Figure 2: Uncropped gel image used in Figure 2D.

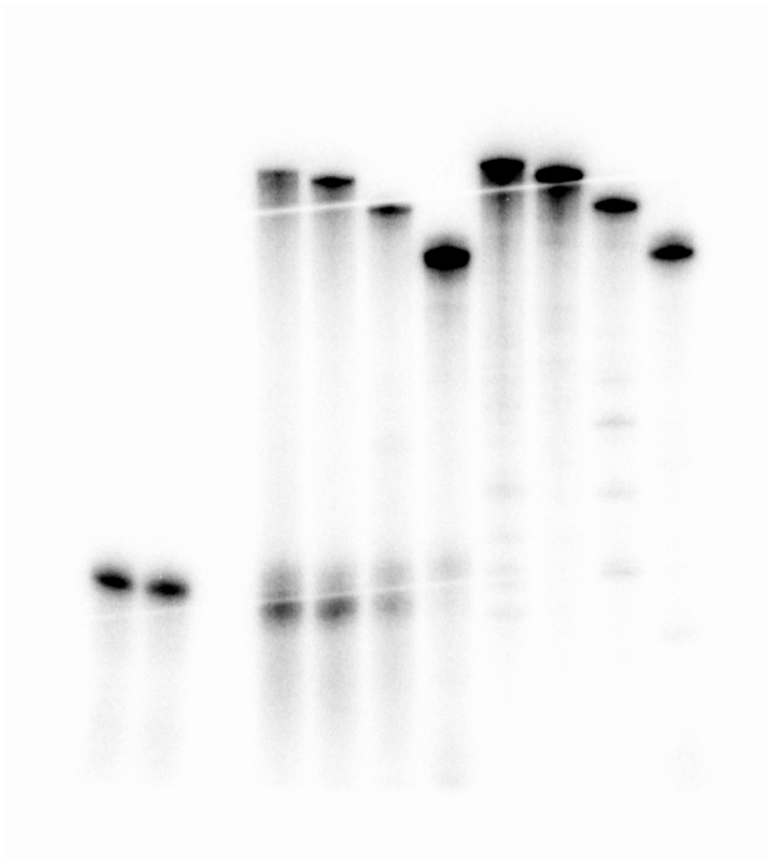

Supplemental Table 1. List of primers used in this study

a) Primers used for synthesizing dsRNA for dsRNA size parameters.

Name	Application	Forward primer sequence (5'-3')	Reverse primer sequence (5'-3')	Fragment size
TuCOPB2_A	RNAi	[T7]-TGGTCGGACAATGGAGATTT	[T7]-TTTTCCTTCGGCATGTATCC	308bp
TuCOPB2_B	RNAi	[T7]-TTCGGGAATCTACAACGTTGC	[T7]-TCAGGTGGAGTATAAACGGCT	513bp
TuCOPB2_100_5'	RNAi	[T7]-AGTTTGTGGTGACGGAGAAT	[T7]-TGAGTCTAGGGCCCAAACAA	97bp
TuCOPB2_200_5'	RNAi	[T7]-AGTTTGTGGTGACGGAGAAT	[T7]-TGCTTCTGGTTTGAATGACGT	181bp
TuCOPB2_400_5'	RNAi	[T7]-AGTTTGTGGTGACGGAGAAT	[T7]-GCTTTGGCAACGTTATCAGG	390bp
TuCOPB2_100_3'	RNAi	[T7]-GCTCGGTCAATAGACTCAATTATTTT	[T7]-TTTTCCTTCGGCATGTATCC	100bp
TuCOPB2_200_3'	RNAi	[T7]-ACGGAATTATTGATGCATTCG	[T7]-TTTTCCTTCGGCATGTATCC	190bp
TuCOPB2_400_3'	RNAi	[T7]-GGAGTTAAATCTGTCGCTGGA	[T7]-TTTTCCTTCGGCATGTATCC	398bp
TuCOPB2_600	RNAi	[T7]-AGTTTGTGGTGACGGAGAAT	[T7]-TTTTCCTTCGGCATGTATCC	612bp
TuVATPase_100_5'	RNAi	[T7]-TCCAACAGTGATGTTATTGTTTACG	[T7]-TAATTGATTCAGTAACTCCATTG	118bp
TuVATPase_200_5'	RNAi	[T7]-TCCAACAGTGATGTTATTGTTTACG	[T7]-ACGGAAATATTCAGATAATGTG	216bp
TuVATPase_400_5'	RNAi	[T7]-TCCAACAGTGATGTTATTGTTTACG	[T7]-ACACTTCCTTCTCTTTCTGGATTAC	413bp
TuVATPase_100_3'	RNAi	[T7]-ACATTTTCCATCCATTAATTGG	[T7]-GAAGAGGTACGAAATCTGGG	97bp
TuVATPase_200_3'	RNAi	[T7]-TCACCACCCGGTGGTGACTTC	[T7]-GAAGAGGTACGAAATCTGGG	196bp
TuVATPase_400_3'	RNAi	[T7]-CCGTGATATGGGTTACCATG	[T7]-GAAGAGGTACGAAATCTGGG	416bp
TuVATPase_600	RNAi	[T7]-TCCAACAGTGATGTTATTGTTTACG	[T7]-GAAGAGGTACGAAATCTGGG	628bp
Control (NC)	RNAi	[T7]-GCCCTCTCCTGGTTGTAAACTT	[T7]-CGACCCCATCAGGCTATTGA	382bp
Chimeric-100_5'-TuVATPase	Chimera	[T7]-TCCAACAGTGATGTTATTGTTTACG	GGAGAGGGCCATTGATTTCAACTGTCAACTC	118bp
Chimeric-NC	Chimera	TTGAAATCAATGGCCCTCTCCTGGTTGTAAAC	[T7]-CGACCCCATCAGGCTATTGA	382bp
T7 promoter sequence		TAATACGACTCACTATAGGG		

b) Primers used for dsRNA synthesis of *Tribolium* orthologs genes in *T. urticae.*

Name	Application	Forward primer sequence (5'-3')	Reverse primer sequence (5'-3')	Fragment size
TuCac	RNAi	[T7]-CGATCGCAATGGTAACTCGC	[T7]-CGTCATCCGACTCTTGTCCC	554bp
TuSrp54	RNAi	[T7]-AGCAGTTACCAACTCTCCCA	[T7]-AGCAGCCCCTGTAATTGTC	685bp
TuRop	RNAi	[T7]-CGAGGCTCCAGAAAAACAC	[T7]-GATGCACTTATCTGCAGGCG	659bp
TuSnap-α	RNAi	[T7]-GATTGTTCGGGGGCTCTTCT	[T7]-TCCCGGGAATCACTGAAAGC	631bp
TuShi	RNAi	[T7]-AAAGCCTCTTTCCTCCGAGC	[T7]-TGTTGGTACGGGTCGTGATG	660bp
TuInr-a	RNAi	[T7]-GGCTCCTACAACCGAACCTC	[T7]-TTCGTTTGCGCTAACTGTGC	664bp
TuHsc70-3	RNAi	[T7]-AACCTACAGCTGCTGCCATT	[T7]-GGGTTAATGCCCCGAGTAGG	568bp
TuRpn7	RNAi	[T7]-AACTGCTGGAGCGTATGAGG	[T7]-AATCTTCGACCCACGCAAGT	639bp
TuGw	RNAi	[T7]-TGACAGCTCCTAGCCAAACA	[T7]-CTCCCATGCCGAATCCCATT	603bp
TuRpt3	RNAi	[T7]-CCTTCAGCTAGTGTCGCCTT	[T7]-CTGGACGGAGTAAAGCAGGG	598bp
TuPp1α-96a_A	RNAi	[T7]-AGAGGTTCGCGGTTCCAAAC	[T7]-TTTGGCGACAACATCAGCAC	652bp
TuPp1α-96a_B	RNAi	[T7]-ATACGGAGGTTTTCCGCCAG	[T7]-ACTGACATCATTCCCCCAGC	624bp

c) Primers used for dsRNA synthesis of a second independent fragment of *Tribolium* orthologs genes in *T. urticae.*

**Name**	**Application**	**Forward primer sequence (5'-3')**	**Reverse primer sequence (5'-3')**	**Fragment size**
TuCac	RNAi	[T7]-AACCCATTCAAACAGGATGC	[T7]-TGGCGTTAAGCCTCCATAAG	612bp
TuSrp54	RNAi	[T7]-GCTGGCCTCAATAAACGAAG	[T7]-ACCACCACCTTTAGCGTGTC	576bp
TuRop	RNAi	[T7]-CGAATGAGCACATCGGATAA	[T7]-GCGCTCAAGAAAGGAAAGTG	569bp
TuSnap-α	RNAi	[T7]-TATCCAGGAAGCCCAGAAAA	[T7]-CAACACACAAATGGCAAAGG	611bp
TuShi	RNAi	[T7]-TGAAACAGACCGAGCAACTG	[T7]-CCCAAACGGTCAACCATATC	510bp
TuInr-a	RNAi	[T7]-CCATGAAGCCATTGGAAACT	[T7]-GTTTGGGTTGCTGGAGGTAA	589bp
TuHsc70-3	RNAi	[T7]-TGATGAAGCTGTTGCCTACG	[T7]-GCACCAAGCTTTTCCTTGTC	582bp
TuRpn7	RNAi	[T7]-CGCTTATTGAGGAAGGTGGT	[T7]-AATCTGGCGAGTTCCATGTC	514bp
TuGw	RNAi	[T7]-TGCACCTATTGCTGCAACTC	[T7]-TCAGGTTTACCCCAAACAGC	672bp
TuRpt3	RNAi	[T7]-TGGTGGTCTCGACATTCAAA	[T7]-TCCTTGCAGTGATGGTTGAG	554bp
TuPp1α-96a_A	RNAi	[T7]-TTGATCCAGCCAATGAACAA	[T7]-TTTCTCCCCATCCCATAACA	657bp
TuPp1α-96a_B	RNAi	[T7]-CACCGACCTTCTGCGATTAT	[T7]-GCTTCGCGAAAAATTCGTAA	575bp

d) Primers used for RT-qPCR with the amplification efficiency of each primer pair.

**Name**	**Gene name**	**Application**	**Forward primer sequence (5'-3')**	**Reverse primer sequence (5'-3')**	**Amplification efficiency**
TuCOPB2	tetur24g00150	RT-qPCR	GTAGAGCCGTGGATGCTCTC	ACACCGAACAGGAACCTCAC	97.51 %
RP49	tetur18g03590 (Ribosomal protein 49)	RT-qPCR	CTTCAAGCGGCATCAGAGC	CGCATCTGACCCTTGAACTTC	102,06 %
CycA	tetur01g12670 (Cyclophilin A)	RT-qPCR	GCTTCAAGGCGGTGACTTT	ACCTGGTCCAGTGTGTTTGAG	102.20 %
